# Cardiovascular patients in COVID‐19 era, a case series, an experience from a tertiary cardiovascular center in Tehran, Iran

**DOI:** 10.1002/ccr3.3163

**Published:** 2020-07-23

**Authors:** Nasim Naderi, Mohammad Mostafa Ansari Ramandi, Mohammadreza Baay, Zahra Hosseini, Mohammad Esmaeil Zanganehfar, Parham Rabieie, Monireh Kamali, Shirin Manshouri, Pardis Moradnejad, Sanaz Asadian

**Affiliations:** ^1^ Rajaie Cardiovascular Medical and Research Center Iran University of Medical Sciences Tehran Iran; ^2^ Cardiovascular Diseases Research Center Birjand University of Medical Sciences Birjand Iran; ^3^ Network of Immunity In Infection, Malignancy And Autoimmunity (NIIMA) Universal Scientific Education And Research Network (USERN) Tehran Iran; ^4^ Cardiovascular Intervention Research Center Rajaie Cardiovascular Medical and Research Center Iran University of Medical Sciences Tehran Iran

**Keywords:** aortic dissection, cardiovascular disorders, coronavirus, myocardial infarction, myocarditis

## Abstract

Different cardiovascular presentations of coronavirus disease 2019 can be seen because of the systemic involvement. Considering its new presentations, there is need for further studies regarding the mechanistic pathways involved.

## Graphical abstract

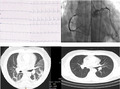

## 1 INTRODUCTION

COVID‐19 (coronavirus disease 2019) has affected more than 4 million people worldwide. Its prominent presentation is respiratory symptoms which can progress to severe pneumonia, respiratory failure and shock; however, many patients may present cardiovascular manifestations.^1^, ^2^, ^3^, ^4^

Although the awareness regarding the adverse impact of cardiovascular involvement on prognosis and the cardiovascular manifestations of COVID‐19 is increasing, the discrimination between the COVID‐19 and non‐COVID‐related cardiovascular etiologies may be challenging.^2^, ^4^, ^5^, ^6^, ^7^, ^8^, ^9^

The concerns about the COVID‐19 may result in a delay in proper approach and prompt management in many emergent and urgent medical conditions, such as cardiovascular problems.

Our center is a tertiary center for cardiovascular medicine in Iran, and as it mainly caters for cardiovascular‐related problems, patients with specific symptoms for COVID‐19 were not referred to our center, because they were transferred to the COVID‐19 specific centers. However, encountering patients infected by the novel coronavirus is inevitable during the pandemic.

In this case series, we report the story of four cases hospitalized in our center with different cardiovascular‐related manifestations at first presentation and suspicious of COVID‐19 after primary workups. Like other centers throughout the world, we aim to present our challenges in the management of these patients and report their final destiny.

## 2 CASE REPORT

### 2.1 Case 1

#### 2.1.1 Case history

A 55‐year‐old man with compressive angina, fever, and dyspnea but no cough and myalgia and reported history of close contact with a COVID‐19‐positive patient is presented. He had a history of hypertension in treatment with captopril and metoprolol succinate.

On admission, he had oxygen saturation (SPO2) of 95%, blood pressure of 100/70 mm Hg, and heart rate of 100 bpm. His temperature was 38 degrees Celsius. The physical examination was unremarkable except for diffuse bilateral crackles.

#### 2.1.2 Differential diagnosis, investigations, and treatment

**FIGURE 1:**
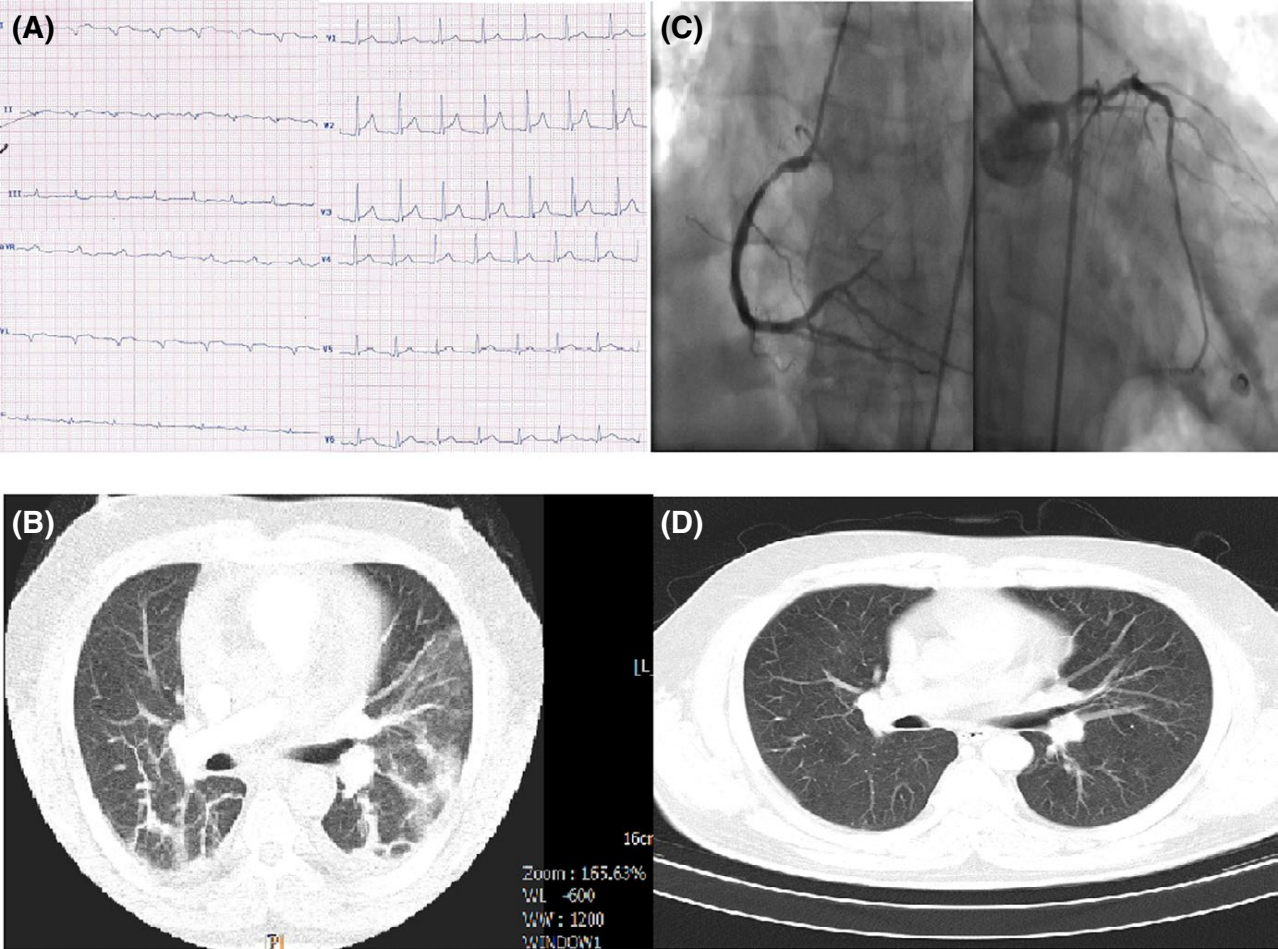
A, ECG of case 1 showing a nonspecific pattern: normal sinus rhythm (NSR), right axis deviation, left atrial abnormality, widening of QRS in limb leads and tall R wave in V1‐V3 as well as ST elevation in V2‐V6. B, Chest CT of case 1 showing typical ground‐glass opacification in both lungs. C, Selective coronary angiography of case 1 revealing three‐vessel disease. D, Chest CT of case 1 after treatment without coronavirus involvement

His electrocardiogram (ECG) showed a nonspecific pattern: Normal sinus rhythm (NSR), right axis deviation, Left atrial abnormality, widening of QRS in limb leads and tall R wave in V1‐V3 as well as ST elevation in V2‐V6 (Figure 1‐Panel A). The main laboratory findings are presented in Table 1. The kidney function tests were normal but liver enzymes were elevated significantly (AST = 269 IU/L and ALT = 276 [normal up to 40]), with normal alkaline phosphatase.

**Table 1:** Important laboratory data of the patients

	Hg mg/dL	WBC count/mL	Lymphocyte count/mL	PMN count/mL	Platelet count/mL	ESR mm/h	hs‐CRP^a^ mg/L	CTn‐I^b^ mcg/L	D‐Dimer^c^ mcg/mL	RT‐PCR for COVID‐19
Case 1	15.2	14 900	1490	12 814	280 000	40	9	2.33	2.33	Positive
Case 2	13.7	9000	801	7974	226 000	13	1	6.39	1.58	Positive
Case 3	9.2	10 300	2060	7725	248 000	90	98	0.19	3.12	Positive
Case 4	14.6	16 610	1063	15 148	229 000	4	30	0.15	–	Negative

Abbreviations: COVID‐19, coronavirus disease 2019; CTn‐I, cardiac troponin I; ESR, erythrocyte sedimentation rate; Hg, hemoglobin; hs‐CRP, high‐sensitivity C‐reactive protein; PMN, polymorphonuclear; RT‐PCR, reverse transcription polymerase chain reaction; WBC, white blood cells.

^a^ Normal value: <6 mg/L.

^b^ Normal value: <0.03 mcg/L.

^c^ Normal value: <0.5 mcg/mL.

Transthoracic echocardiogram (TTE) showed normal LV size, left ventricular ejection fraction (LVEF) of about 45% without RWMA, a grade one of LV diastolic dysfunction and normal pulmonary artery pressure (PAP). The first chest computed tomography (CT) showed typical ground‐glass opacification (GGO) pattern in both lungs (Figure 1‐Panel B).

He was admitted in monitored bed at the COVID‐specific ward, and nasopharyngeal swab specimen was taken for reverse transcription polymerase chain reaction (RT‐PCR) for COVID‐19, which was reported positive the following day. At the second day of admission, his symptoms in terms of dyspnea aggravated, he developed dry cough, and his O2 saturation dropped to 75% without O_2_ therapy. He received hydroxychloroquine (HCQ) for 5 days at first line, but the pneumonitis was not controlled; thus, lopinavir/ritonavir (Kaletra) started for him by an infectionist, leading to improvement of his symptoms and SPO2.

He was ready for discharge after a negative RT‐PCR, 2 days after the second chest CT; however, he developed severe angina with identical ECG changes and a CTn‐I of >50 µg/L; thus, the patient was emergently transferred to catheterization laboratory (Cath‐Lab), where his selective coronary angiography revealed three‐vessel disease (Figure 1‐Panel C).

#### 2.1.3 Outcome

Coronary bypass surgery was planned for the patient, but he refused and got better after intensification of antiangina therapies, echocardiography before discharge was not significantly different from his baseline echo except for a mild decrease in left ventricular function (LVEF = 35%‐40%) and he was discharged after about 2 months of hospitalization. His antihypertensive medications were continued during his hospitalization course. Figure 1‐panel D shows his chest CT, 35 days after completion of therapies which depicts that the pneumonitis is fully resolved. The ECG changes were resolved, and the CTn‐I returned to normal.

### 2.2 Case 2

#### 2.2.1 Case history

A 61‐year‐old man with acute‐onset angina chest pain without fever, a sore throat, dyspnea, cough, myalgia, and no history of close contact with a COVID‐19‐positive patient is presented. He had past history of hypertension, smoking, and chronic obstructive pulmonary disease. He was addicted to opium and had a recent history of percutaneous coronary intervention (PCI) on left anterior descending (LAD) artery and right coronary artery (RCA) 2 months before. He was under treatment with valsartan, aspirin, bisoprolol, furosemide, spironolactone, nitroglycerin, clopidogrel, and atorvastatin.

On presentation, he had SPO2 = 94%, blood pressure = 140/80 mm Hg, heart rate = 90, and temperature = 37. The physical examination was unremarkable save diffuse bilateral crackles, and systolic murmur at apex and left sternal border.

#### 2.2.2 Differential diagnosis, investigations, and treatment

**FIGURE 2:**
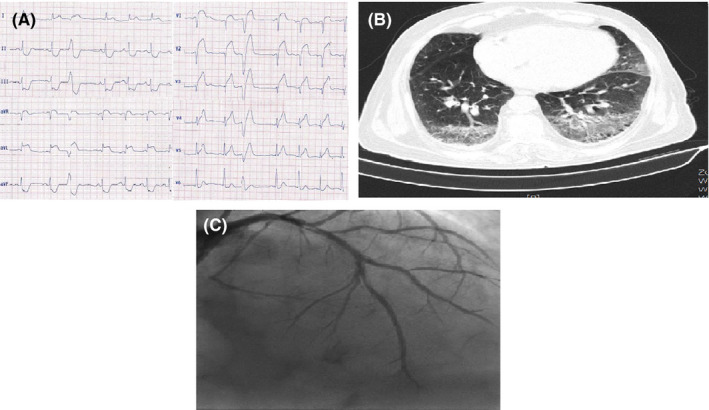
A, ECG of case 2 showing ST‐segment elevation in anterior precordial leads. B, Angiographic view of case 2 showing stent thrombosis in LAD. C, Chest CT of case 2 with typical findings for coronavirus infection

His electrocardiogram (ECG) showed ST‐segment elevation in anterior precordial leads (Figure 2‐panel A).The main laboratory findings are presented in Table 1. The kidney and liver function tests were normal. The diagnosis was acute anterior ST‐elevation myocardial infarction (MI), and he was emergently transferred to the Cath‐Lab and underwent primary PCI on LAD due to stent thrombosis (Figure 2‐panel B). He received Integrilin after the procedure with a dose of 2 µg/kg/min for 48 hours.

Echocardiography just after primary PCI revealed normal LV size, LVEF = 25%, anterior wall akinesia, severe hypokinesia of anterolateral, and lateral walls, grade I LV diastolic dysfunction, moderate mitral regurgitation (MR), and normal PAP.

The next day, he developed dry cough and his chest CT examination was typical for COVID‐19 (Figure 2‐panel C). He was transferred to the COVID‐19‐specific monitored bed, and the nasopharyngeal swab specimen was positive for COVID‐19 in RT‐PCR test.

He also received HCQ for 5 days accompanied with his cardiac medications standard for MI. The disease course was uneventful, the ECG changes improved, and the LVEF increased to 35%.

#### 2.2.3 Outcome

He was discharged with self‐consent, having decent clinical conditions on discharge without chest pain or cough. He was advised to be isolated, and all family members were educated. The patient had good clinical condition upon phone follow‐up.

### 2.3 Case 3

#### 2.3.1 Case history

A 61‐year‐old lady with nausea, vomiting, weakness, dyspnea, and orthopnea was a known case of hypertension, diabetes mellitus, and end‐stage renal disease. She had also a right kidney mass diagnosed about 2 months before and was under workup. She had been under hemodialysis 3 days per week and also had a history of permanent pacemaker implantation 2 months before her last admission due to complete heart block. She was on statins, insulin, and calcitriol.

On physical examination, she had SPO2 = 82%, which improved to 96% with nasal O2, blood pressure = 90/60 mm Hg, and heart rate = 100 bpm and no fever. No other remarkable finding was noted.

#### 2.3.2 Differential diagnosis, investigations, and treatment

**FIGURE 3:**
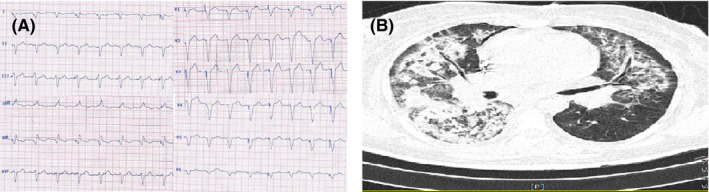
A, ECG of case 3 showing pacemaker's rhythm. B, Chest CT of case 3 showing bilateral confluent consolidations predominantly in upper and middle zones with more severity in right side

Electrocardiogram showed pacemaker's rhythm (Figure 3‐panel A). The baseline laboratory findings are presented in Table 1. Renal function test was severely disturbed (creatinine = 5.4 mg/dL), and she had also hyponatremia (serum sodium = 126 meq/dL) and mild hyperkalemia (serum potassium = 5 meq/dL). The baseline liver function test was within normal limits.

Emergent limited bedside TTE at emergency department (ED) showed massive pericardial effusion (PE) with a maximal depth of 3 cm and evidence of cardiac tamponade. She was urgently transferred to the operating room for the PE drainage.

Postdrainage TTE showed moderate left ventricular enlargement, LVEF = 30%, moderate‐to‐severe MR, mild‐to‐moderate tricuspid regurgitation, a PAP of 45 mm Hg, and small PE. The current echocardiography showed a significant reduction in her LV function considering her previous TTE report (2 months earlier), which was completely within normal limits.

After surgery, the extubation was impossible due to the respiratory distress and development of hypoxemia during the weaning process and she got febrile.

Chest CT showed bilateral confluent consolidations predominantly in upper and middle zones with more severity in right side highly suggestive of COVID‐19 (Figure 3‐panel B).

She was isolated in ICU, and the nasopharyngeal swab specimen was reported positive for COVID‐19 in RT‐PCR test.

#### 2.3.3 Outcome

The patient's status deteriorated, and she underwent shock and unfortunately died after about 50 days. She received Kaletra and IVIG to control COVID‐19 infection, accompanied with vasopressor support and hemodialysis.

The pericardial fluid analysis was compatible with an exudate nature with a negative cytology. The pericardial biopsy was negative for malignancy and tuberculosis.

### 2.4 Case 4

#### 2.4.1 Case history

The patient is a 22‐year‐old healthy man with compressive chest pain, dyspnea, and back pain without a sore throat, dyspnea, and cough and had no history of close contact with a COVID‐19‐positive patient. He was a nonsmoker, and his past medical and drug history were negative.

On presentation, he had SPO2 = 93%, systolic blood pressure of upper extremities was about 60 mm Hg, and the BP of lower extremities was 140/60 mm Hg. His heart rate was 90 bpm, and temperature was 38.1 degree Celsius. The physical examination was unremarkable except for impalpable pulse of left upper limb and weak pulse of right upper limb. He did not have any sign of Marfan syndrome.

#### 2.4.2 Differential diagnosis, investigations, and treatment

**FIGURE 4:**
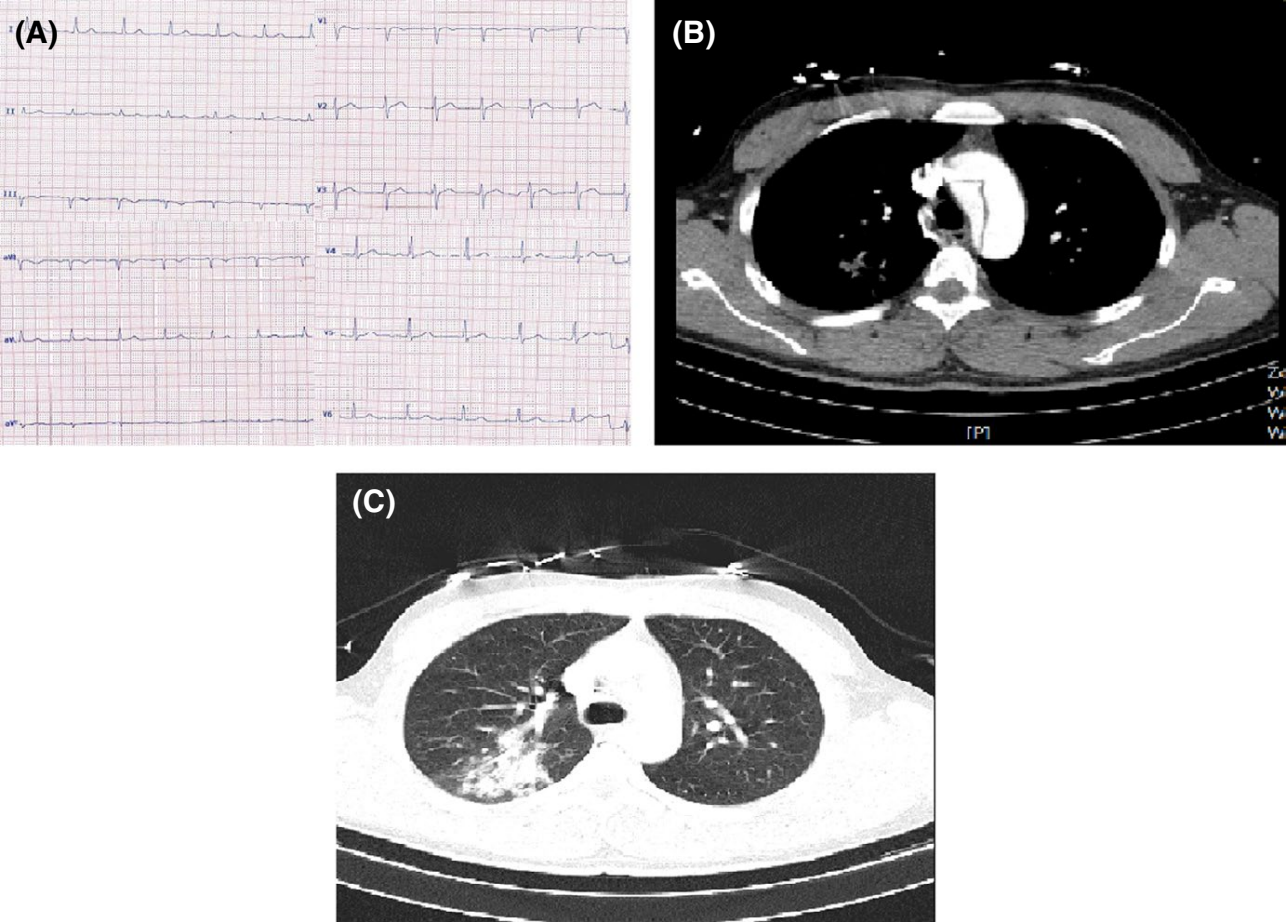
A, Normal ECG of case 4. B, Aortic CT angiography of case 4 showing tear in aorta. C, Chest CT of case 4 showing multifocal confluent round‐shaped consolidations in left lower lobe mostly compatible with COVID‐19

His ECG was within normal limits (Figure 4‐panel A). Emergent TTE showed a normal LV size with LVEF of 50%‐55%, normal RV size and function, mild MR, tricuspid aortic valve and moderate aortic insufficiency, no aortic stenosis, and dilated ascending aorta (5.1 cm); flap of dissection was seen in ascending aorta originating from sinotubular junction and extending to aortic arch, descending aorta and abdominal aorta. The left subclavian artery originated from the false lumen. No PE was observed. The laboratory data are presented in table 1.

The patient was emergently transferred to CT department where chest CT, aortic CT angiography, and coronary angiography were performed for him.

Coronary CT angiography showed normal coronary arteries.

Aortic CT angiography showed type A aortic dissection. The intimal flap started at distal part of the ascending aorta with extension to abdominal aorta and terminated distal to the renal arteries. Dissection flap extension to brachiocephalic artery, left common carotid and left subclavian artery were noted. Both true and false lumens were patent. Distal abdominal aorta and iliac arteries were intact (Figure 4‐panel B).

Chest CT showed multifocal confluent round‐shaped consolidations in left lower lobe mostly compatible with COVID‐19 (Figure 4‐panel C).

The laboratory findings are presented in Table 1. The renal and liver function tests were normal.

The patient was scheduled for emergent surgery for acute aortic dissection, but after being informed of the potential COVID‐19, he and his family refused the surgery in our center and asked for transfer to a COVID‐19 dedicated center with cardiac surgery facilities. Despite explaining that he may be endangered during transfer, he was transferred to another center by a fully equipped ambulance after taking signed self‐informed consent.

#### 2.4.3 Outcome

The phone follow‐up on the following days revealed that he had undergone cardiac surgery successfully. We could not get access to his further clinical course and medical therapies, but our further phone follow‐up revealed that he was discharged from hospital after several weeks with good conditions. However, the first RT‐PCR of nasopharyngeal swab test sent by our center was negative.

## 3 DISCUSSION

Case 1 was initially manifested with cardiac‐related symptoms and signs and developed a typical form of COVID pneumonitis later. This patient had a history of hypertension, which is one of the very important comorbidities and risk factors for progression of COVID‐19 into its severe forms. Although his illness rapidly progressed and the lung involvement was significant, and despite history of HTN, he did not progress to acute respiratory distress syndrome or shock and responded well to the therapies.

We continued his ACEI (captopril) to control his blood pressure and due to its beneficial effects on his LV function during his disease course and on discharge.

Regardless of many clinicians' concerns about susceptibility to coronavirus in those patients who are taking ACEIs or ARBS, observational studies have not provided convincing data on whether these drugs have potentially beneficial or harmful effects on COVID‐19 patients. It has been shown that worse outcomes may be more prevalent in hypertensive and diabetic patients, possibly due to overexpression of angiotensin‐converting enzyme 2 (ACE‐2) receptors in alveolar epithelial cells. Some researchers speculate that as using ACEIs and ARBs upregulate ACE‐2 expression, theoretically this effect could increase the virus entry and risk for COVID‐19 or disease severity. In contrast, some investigators have postulated some mechanisms for the beneficial effects of these drugs. To exemplify, upregulating ACE2 with ACEI or ARBs could enhance the conversion of angiotensin II to angiotensin 1‐7 which has anti‐inflammatory and vasodilatory properties. Nevertheless, professional societies such as ACC/AHA have recommended that patients receiving these drugs should continue taking them.^3^, ^8^, ^10^, ^11^, ^12^, ^13^

Decision‐making under uncertainty would be stressful in such settings. However, we decided to continue the ACEI because we postulated that stopping the drug in a compensated heart failure patient might be more harmful.^3^, ^14^, ^15^

Another issue regarding the current patient is the development of non‐ST elevation MI (NSTEMI) after controlling his pneumonitis. As shown earlier, the endothelial dysfunction in the setting of coronavirus infection may lead to the plaque instability, microvascular dysfunction, and thrombus formation in previously diseased coronary arteries. On the other hand, prolonged hypoxemia in this patient could be considered a predisposing factor for MI development.

The second case is another example of an acute thrombotic event as the first presentation of COVID‐19. Although he was in his first 3 months after coronary angioplasty, in which the risk of acute thrombosis is high, an increased risk for the stent thrombosis by COVID‐19 could not be simply ruled out.^3^, ^14^, ^16^, ^17^

There are some reports regarding the early stent thrombosis in COVID‐19 patients with acute MI patients having undergone primary PCI. To the best of our knowledge, this case may be the first reported case of in‐stent thrombosis in a COVID‐19 patient with history of chronic PCI.

The mechanism of increased risk of stent thrombosis may be the increased platelets' aggregability accompanied with other pathophysiological mechanisms mentioned earlier, such as endothelial dysfunction.

For the third case, although patients who are under hemodialysis are predisposed to uremic pericarditis and tamponade, this patient did not reveal any history of missing her routine hemodialysis. Hua et al have reported the first case of tamponade complicating myopericarditis in a 47‐year‐old lady.^18^ Considering the clinical and laboratory findings of the current patient, it seems that our patient is another case of COVID‐19 myopericarditis.

The fourth case shows the development of aortic dissection in an otherwise normal individual probably infected with novel coronavirus. Although the RT‐PCR for novel coronavirus was negative, the other laboratory findings and the chest CT were highly suggestive of COVID‐19.

Fukuhara et al have reported an acute type A aortic dissection in a healthy nonsmoker man who initially did not have any signs of COVID‐19, and on the 5th day after a successful surgery, he developed bilateral lung infiltrate which progressed to ARDS and death and a positive RT‐PCR for COVID‐19.^19^ They did not consider the COVID‐19 the possible cause of aortic dissection and aimed to highlight the importance of being always ready to encounter COVID‐19 in any patient with any complaint and to assume all patients may have been exposed to COVID‐19. However, the novel coronavirus has surprised the clinicians and investigators since its first appearance. It can invade everywhere in the body, and in these cases, large vessel arteritis could not be simply ruled out.

## CONFLICT OF INTEREST

None declared.

## AUTHOR CONTRIBUTIONS

NN and MMAR: contributed to acquisition of data, drafting the manuscript, final revision of the manuscript, and participated sufficiently in the work. MB, ZH, MEZ, PR, MK, SM, PM, and SA: contributed to acquisition of data, drafting the manuscript, and participated sufficiently in the work.

## ETHICAL APPROVAL

Informed consent was acquired from patients to report their data. The ethical committee of Rajaie Cardiovascular Medical and Research Center approved this case series.
